# Supplementary material for: Pain-Free Alpha-Synuclein Detection by Low-Cost Hierarchical Nanowire Based Electrode
Source: Nanomaterials (Basel). 2024 Jan 12;14(2):170. doi: 10.3390/nano14020170 (PMC10819810; doi:10.3390/nano14020170)
Supplement: Supplementary file 1 [file nanomaterials-14-00170-s001.zip › nanomaterials-2809127-supplementary.pdf]

nanomaterials

Supplementary Materials

Pain-Free Alpha-Synuclein Detection by Low-Cost Hierarchical Nanowire Based Electrode

Gisella M. Di Mari [1,2], Mario Scuderi [3], Giuseppe Lanza [4,5], Maria Grazia Salluzzo [5], Michele Salemi [5], Filippo Caraci [5,6], Elena Bruno [1,2], Vincenzina Strano [2], Salvo Mirabella [1,2] and Antonino Scandurra [1,2,7,*]

[1] University of Catania, Department of Physics and Astronomy, "Ettore Majorana", via S. Sofia 64, 95123 Cata-nia, Italy; gisella.dimari@dfa.unict.it; elena.bruno@dfa.unict.it; salvo.mirabella@dfa.unict.it

[2] CNR-IMM, Catania (University) UNIT, via S. Sofia 64, 95123 Catania, Italy; vincenzina.strano@ct.infn.it

[3] CNR-IMM, VIII Strada 5, 95121, Catania, Italy; mario.scuderi@imm.cnr.it;

[4] University of Catania, Department of Surgery and Medical-Surgical Specialties, Via Santa Sofia 78, 95123, Catania, Italy; giuseppe.lanza1@unict.it

[5] Oasi Research Institute-IRCCS. Via Conte Ruggero 73, 94018, Troina, Italy; msalluzzo@oasi.en.it; msalemi@oasi.en.it

[6] University of Catania, Department of Drug and Health Sciences, Via Santa Sofia 64, 95123, Catania, Italy; fcaraci@unict.it

[7] Research Unit of the University of Catania, National Interuniversity Consortium of Materials Science and Technology (INSTM-UdR of Catania), via S. Sofia 64, 95125 Catania, Italy

* Correspondence: Correspondence: antonino.scandurra@dfa.unict.it

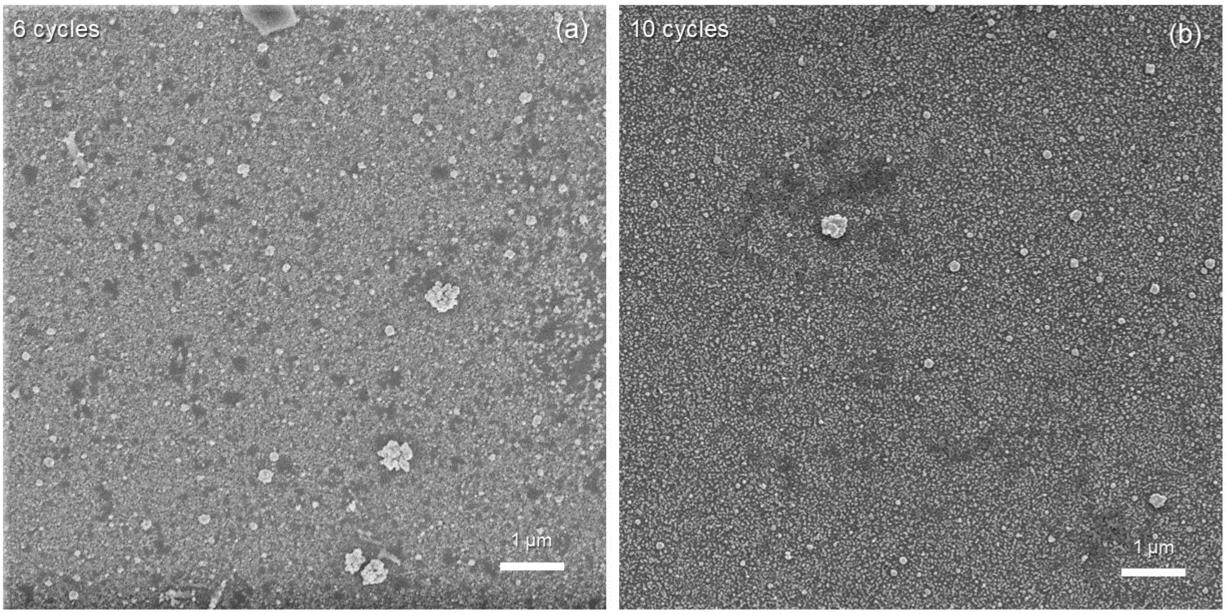

Figure S1. FESEM pictures of Au NPs obtained through electrodeposition, performing a) 6 CV cycles and b) 10 CV cycles, respectively.

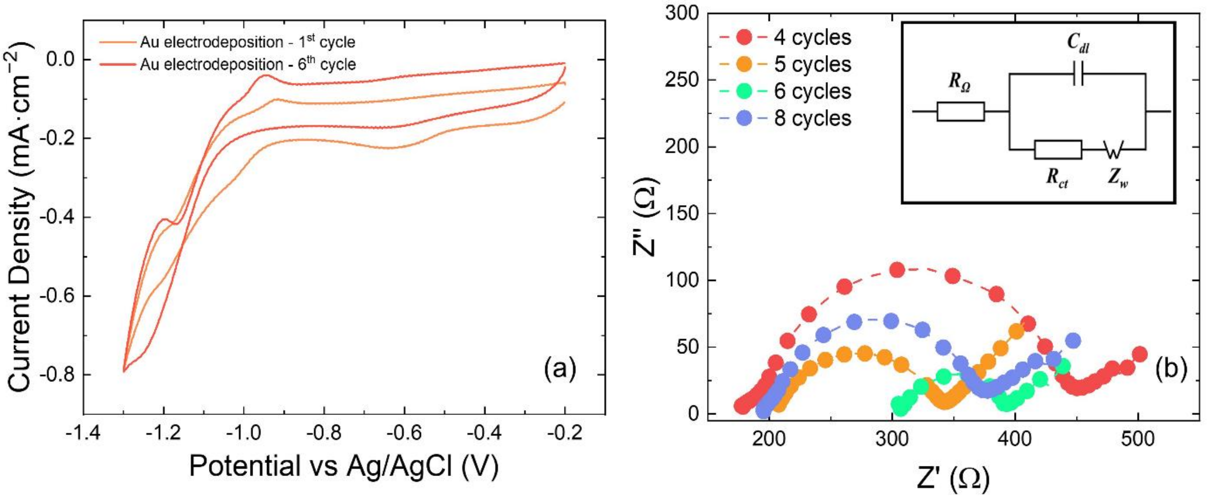

Figure S2. (a) Au electrodeposition first and sixth cycles; the electrodeposition was conducted in a 1mM $HAuCl_4$ solution in PBS by performing cyclovoltammetries, by sweeping the potential from -0.2V to -1.3V with a scan rate of 50 mV s^{-1}. (b) Experimental (dots) and simulated (dashed lines) impedance spectra (EIS) in the frequency range of 0.2 to $5{\cdot}10^4$ Hz of Au NPs into ITO-PET, obtained at a formal potential of 0V. In particular, 4 (red), 5 (orange), 6 (mint), 8 (blue) cycles were evaluated. Inset: equivalent electrical circuit used for the EIS simulations.

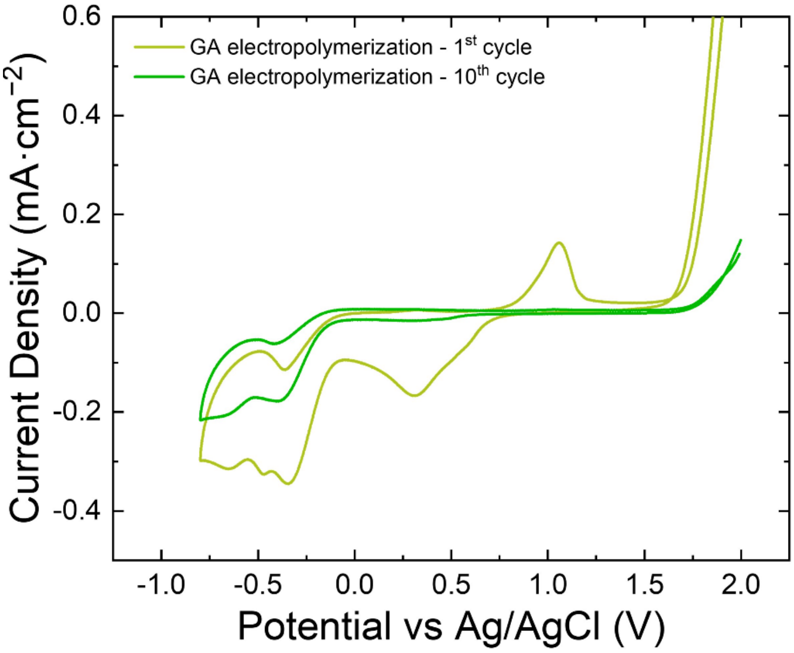

Figure S3. First and tenth CV cycles of GA electro-polymerization. The electro-polymerization was performed by cyclic voltammetry in a L-glutamic acid solution 0.01M in PBS, by executing 10 cycles in the potential range of –0.8V to 2V.

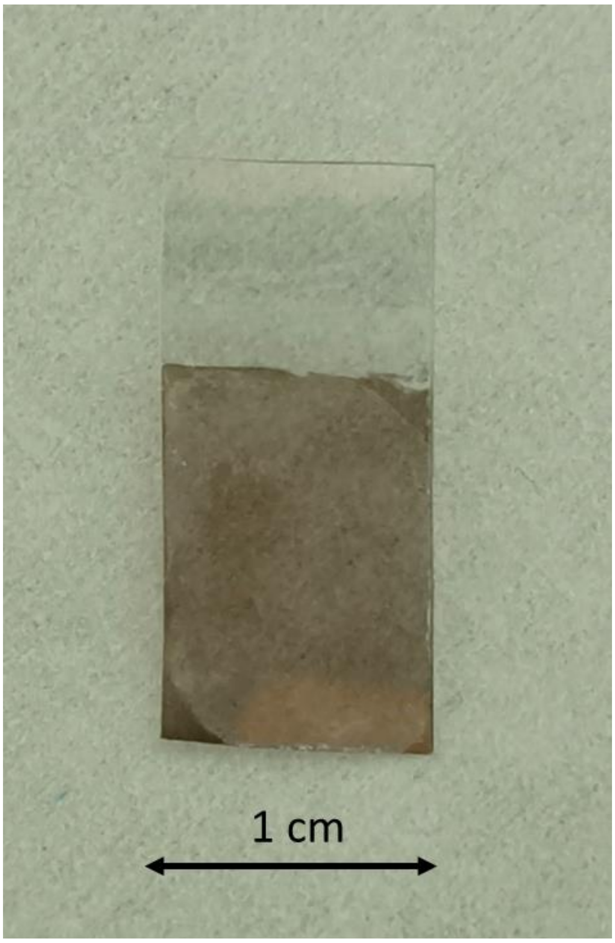

Figure S4. Optical picture of the whole electrode.

Electrochemical tests before and after α-synuclein immobilization

Figures S5 and S6 shows the CV curves and the experimental (dots) and simulated dashed lines) impedance spectra (EIS) in the frequency range of 0.2 to $5 \cdot 10^4$ Hz of four electrodes (S1-S4) before (empty circles) and after the α-synuclein immobilization step (full circles), at concentration of 0.5, 1, 3.5, and 10 $pg \cdot mL^{-1}$ in PBS solution. As a consequence of α-synuclein immobilization, the electrode was subjected to an increase in terms of R_{ct}. Similarly, both the anodic and cathodic peaks of the probe redox couple were subjected to a reduction in the current density as a function of the α-synuclein concentration and to a shift towards higher potentials, as reported in Figure S5.

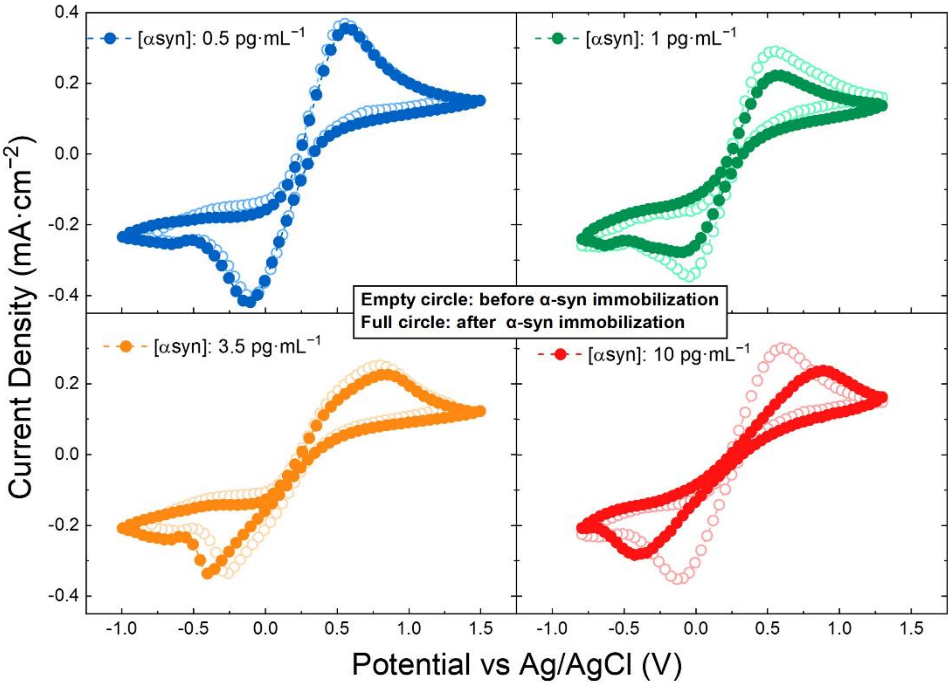

Figure S5. Cyclic voltammograms of the redox probe $Fe(II)(CN)_6^{4-}/Fe(III)(CN)_6^{3-}$ recorded before (empty circle) and after α-synuclein immobilization (full circle) at concentrations of 0.5, 1, 3.5 and 10 pg·mL^{-1} in PBS solution.

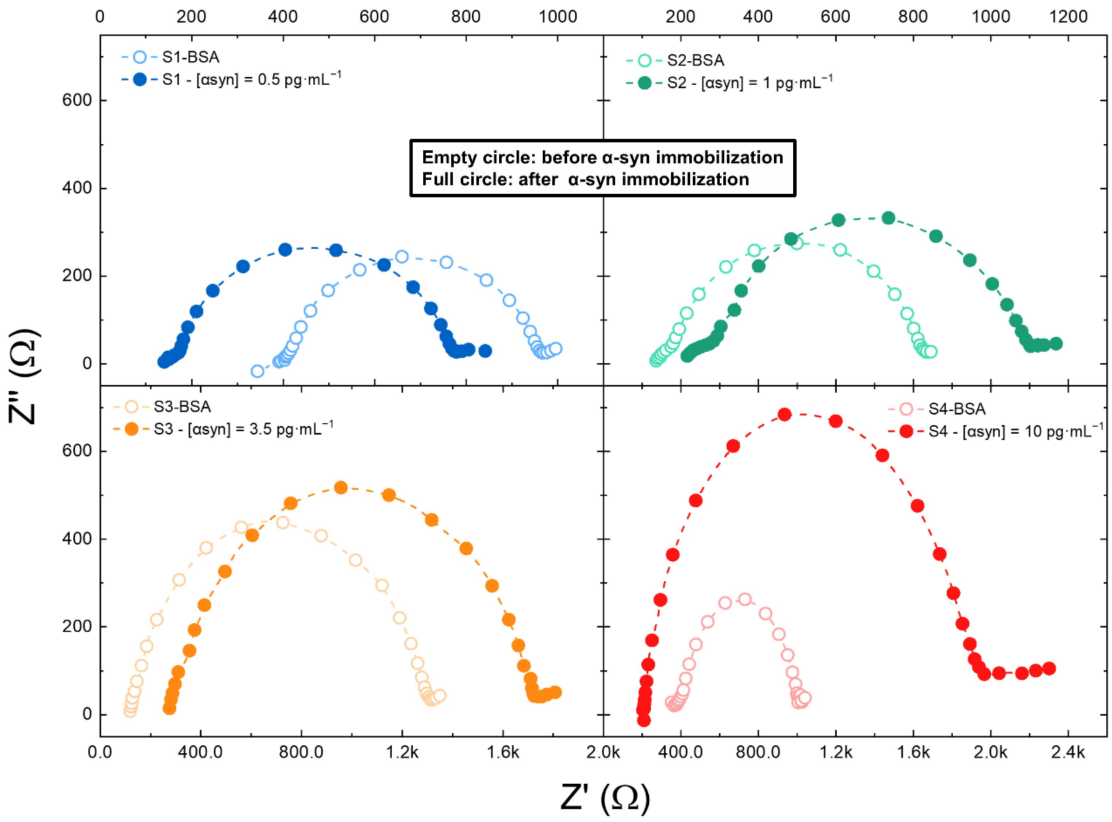

Figure S6. Experimental (dots) and simulated (dashed lines) impedance spectra (EIS) in the frequency range of 0.2 to 5·10^4 Hz of the electrodes recorded before (empty circles) and after α-synuclein recognition and immobilization (full circles), at concentrations of 0.5, 1, 3.5 and 10 pg·mL^{-1} in PBS solution.
